# Inhibition of angiogenetic macrophages reduces disc degeneration-associated pain

**DOI:** 10.3389/fbioe.2022.962155

**Published:** 2022-10-11

**Authors:** Yang Hou, Jiangang Shi, Yongfei Guo, Guodong Shi

**Affiliations:** Department of Orthopaedic Surgery, Changzheng Hospital, Second Military Medical University, Shanghai, China

**Keywords:** macrophage polarization, vascular endothelial growth factor A (VEGF-A), angiogenesis, innervation, lumbar disc degeneration (LDD)

## Abstract

Abnormal angiogenesis and innervation in avascular discs during lumbar disc degeneration (LDD) cause severe back pain. These pathological alterations in the degenerating discs are induced by cytokines partially produced and secreted by inflammatory cells, among which macrophages are the most frequently ones detected at the legion site. However, the role of macrophages as well as their polarization in regulation of innervation and angiogenesis in the degenerating discs is unclear. In this study, we analyzed macrophages in the degenerating discs from patients and detected a specific macrophage subtype that expresses high levels of vascular endothelial growth factor A (VEGF-A). Co-expression of M2 macrophage markers in this macrophage subtype suggested that they were a M2d-like subtype. High levels of VEGF-A and genes associated with angiogenesis were also detected in LDD specimens compared to control heathy discs from a public database, consistent with our finding. Moreover, the levels of VEGF-A in disc macrophages were strongly correlated to the pain score of the examined patients, but not to the Thompson classification of the degeneration level of the patients. *In vitro*, overexpressing VEGF-A in macrophages increased the tube formation, proliferation and migration of co-cultured endothelial cells, and increased the innervation of embryonic spinal cord explant into the co-cultured area for macrophages and skeletal myocytes. *In vivo*, an orthotopic injection of adeno-associated virus carrying siRNA for VEGF-A under a macrophage-specific CD68 promoter significantly reduced the number of VEGF-A-positive disc macrophages and alleviated the pain in LDD-mice. Together, these data suggest that inhibition of angiogenetic potential of macrophages may reduce disc degeneration-associated pain through suppression of angiogenesis and innervation, as a promising therapy for LDD-associated pain.

## Introduction

A normal intervertebral disc (IVD) consists of an outer layer of annulus fibrosus (AF), a central layer of nucleus pulposus (NP) and cartilage end plates in between (Kamali et al., 2021). The structure of IVD is avascular, maintained by proteoglycans that inhibit the growth of nerve and blood vessels into the disc (Dickinson and Bannasch, 2020). Thus, IVD acquires nutrients from outside vessels by diffusion through the extracellular matrix (Risbud and Shapiro, 2014). During lumbar disc degeneration (LDD), disc vascularization and innervation occur secondary to loss of proteoglycans and water content, leading to pain development (Risbud and Shapiro, 2014).

It is believed that disc vascularization and innervation may be induced by inflammatory cytokines, which are partially produced and secreted by inflammatory cells (Le Maitre et al., 2015). Past studies have also shown that vascular endothelial growth factor A (VEGF-A) and its receptor signaling play a critical role in the disc vascularization (David et al., 2010). The sources of VEGF-A in the degenerating discs include AF, NP, endothelial cells in the newly formed vessels and inflammatory cells (Binch et al., 2014). However, the degree of the contribution of these cells to the total disc VEGF-A levels should be dynamic and the pattern has not been fully determined so far. Moreover, it is not known which cells produce VEGF-A at beginning to initiate the pathological changes.

Among all the inflammatory cells, macrophages are the most frequently ones detected at the legion site (Yamagishi et al., 2022; Yamamoto et al., 2022). Macrophages are important cells in innate immune response and traditionally believed as phagocytotic cells to eliminate foreign pathogens and dead cells in the body (Thiriot et al., 2020). However, many additional functions of macrophages were detected later on (Yunna et al., 2020). The classical phagocytotic macrophages were then termed “M1”, while macrophages of alternative polarization (the differential status) associated with repair, regeneration and remodeling are termed “M2” (Wang et al., 2021). M2 macrophages were further separated into 4 main groups as M2a, M2b, M2c, and M2d, among which M2a represents trophic M2 macrophages (Ferrante and Leibovich, 2012), M2b represents M2 macrophages more associated with inflammation (Ferrante and Leibovich, 2012), M2c represents M2 macrophages expressing high transforming growth factor beta 1 (TGFβ1) that induces fibrosis (Lawrence and Natoli, 2011), and M2d represents M2 macrophages expressing high VEGF-A to be angiogenetic (Wang et al., 2010; Nikovics et al., 2020). Interestingly, we have previously shown that epigenetic regulation of macrophage polarization and specific inhibition of TGFβ1 alleviate the severity of LDD (Hou et al., 2020). However, the role of macrophage polarization in the regulation of innervation and angiogenesis as well as development of the pain in the degenerating discs is unclear and thus addressed in this study.

## Materials and methods

### Protocol approval and animal work

This study was approved by the Research and Animal Ethics Association of Second Military Medical University. Balb/c mice (Animal Laboratory of the Academy of Medical Sciences, Beijing, China) were used for LDD induction and related experiments at age of 12 weeks. Male and female mice were evenly distributed in 4 experimental groups of 5 each. Group 1, mice received sham operation and intradiscal injection of empty solvent of equal volume with a 31-G needle (Sham); Group 2, mice received LDD induction by surgical removal of the spinal muscles, ligaments from supraspine and intraspine and posterolateral halves of the bilateral zygapophysial joints (Hou et al., 2020) and intradiscal injection of empty solvent of equal volume with a 31-G needle (LDD); Group 3, mice received LDD induction and simultaneous intradiscal injection of 100 µl 10^11^ scramble-AAVs with a 31-G needle (LDD+Scr); Group 4, mice received LDD induction and simultaneous intradiscal injection of 100 µl 10^11^ si-VEGF-A-AAVs with a 31-G needle (LDD+si-VEGF-A). Mice were analyzed 4 weeks after treatments, since it is short enough to maintain the transgenes from AAVs but long enough to allow the biological effects to occur secondary to the treatment with AAVs. A von Frey filament test was performed as described before (Hou et al., 2020). Briefly, the mice were placed in a test box, from where Von Frey microfilaments were used to press the hind paw on the side of the mice to cause motionless withdrawal of its hind leg.

### Cells, explants and AAVs

Interleukin 4 (IL-4; 20 ng/ml, Invitrogen, Shanghai, China) was used to prime mouse bone marrow derived macrophages (BMDM) to M2 phenotype. All cell lines and primary cells were cultured in at 37°C, in a 5% CO_2_. BMDM were isolated and cultured in Dulbecco’s modified Eagle medium (DMEM; Invitrogen) with 8% fetal bovine serum (FBS, Sigma-Aldrich, Beijing, China). A mouse endothelial cell line MS1 was purchased from American Type Culture Collection (ATCC, Rockville, MD, United States) and cultured in DMEM with 10% FBS. Skeletal myocytes were isolated from gastrocnemius muscle from Balb/c mice with a 2.5 h’ digestion with 2 mg/ml Collagenase I (C0130, Sigma-Aldrich, Shanghai, China), followed by a selection culture as described (Beauchamp et al., 2000). Spinal cord culture used ventral horn free of meninges and root ganglia from day 12 embryonic mouse spinal cords from Balb/c mice. Serotype 2 was chosen for AAV generation from transfecting human embryonic kidney 293 cells with prepared transgenes, VEGF-A, scramble, and si-VEGF-A, respectively. The complete VEGF-A coding sequence was obtained from PCR with cDNA from human 293 cells. Sequences for scramble and si-VEGF-A are 5′-GGT​ATC​TAC​TAG​ATG​TAC​T-3′ and 5′-TGT​GAA​TGC​AGA​CCA​AAG​A-3′, respectively. Human CD68 promoter was obtained from Addgene (#34837, Addgene, Watertown, MA, United States) (Lang et al., 2002). A multiplicity of infection (MOI) of 100 was used to transduce macrophages *in vitro*.

### Co-culture system

A transwell system (8 μm pore size, Corning Co., NY, United States) was used for co-culture of endothelial cells (ECs) with macrophages. A density of 200 μl of 10^5^ cells/ml was used for each chamber. A microfluidic chamber system was used for assessing innervation of spinal cord explant into area of co-culture of skeletal myocytes and macrophages, as described (Gluska et al., 2014). Briefly, the explant was well positioned, after which the device was attached to a 60-mm plastic dish with margins sealed with polydimethylsilxane (PDMS) at 60°C for 30 min to prevent the chamber from detaching. Muscle channels were then coated with Matrigel diluted 1:10 with DMEM. The explant well and channel were sequentially filled with 150 ml of 1.5 ng/ml polyornithine (P-8638, Sigma-Aldrich) in PBS and 150 ml laminin (L-2020, Sigma-Aldrich) each for 12 h, respectively. The co-culture was then started afterwards after replacement with corresponding media.

### Flow cytometry

For flow cytometry analysis, digested single cell fractions were fixed with 10% formalin for 15 min, then labeled with PE-conjugated CD68, PE-cy7-conjugated CD206, FITC-conjugated arginase 1 and BV421-conjugated VEGF-A antibodies (Becton-Dickinson Biosciences, Shanghai, China). Flow cytometry data were analyzed and presented with FlowJo software (Flowjo LLC, Ashland, OR, United States).

### Immunocytochemistry and ELISA

In cultured cells, GFP was detected by direct fluorescence. ELISA was performed using kits for VEGF-A (R&D System, Los Angeles, CA, United States).

### Real-time quantitative polymerase chain reaction

RNA extraction, cDNA synthesis and RT-qPCR were performed using all reagents from Qiagen (Shanghai, China), including all RT-qPCR primers. GAPDH was proved to be stable across samples and thus used as a housekeeping gene for normalizing the expressing values of examined genes.

### Assessment of lumbar disc degeneration degrees

Histological determination of LDD degrees was done using the method by Melgoza et al. (2021).

### Statistical analysis

All statistical analyses were carried out using GraphPad prism (GraphPad Software, Inc. La Jolla, CA, United States). One-way ANOVA with a Bonferroni correction, followed by Fisher’s exact test was applied. Data were presented as mean ± standard deviation (SD) and are considered significant if *p* < 0.05. No significance (ns) was considered when *p* > 0.05.

## Results

### Detection of a vascular endothelial growth factor A-expressing disc macrophage subtype in lumbar disc degeneration patients

**FIGURE 1:**
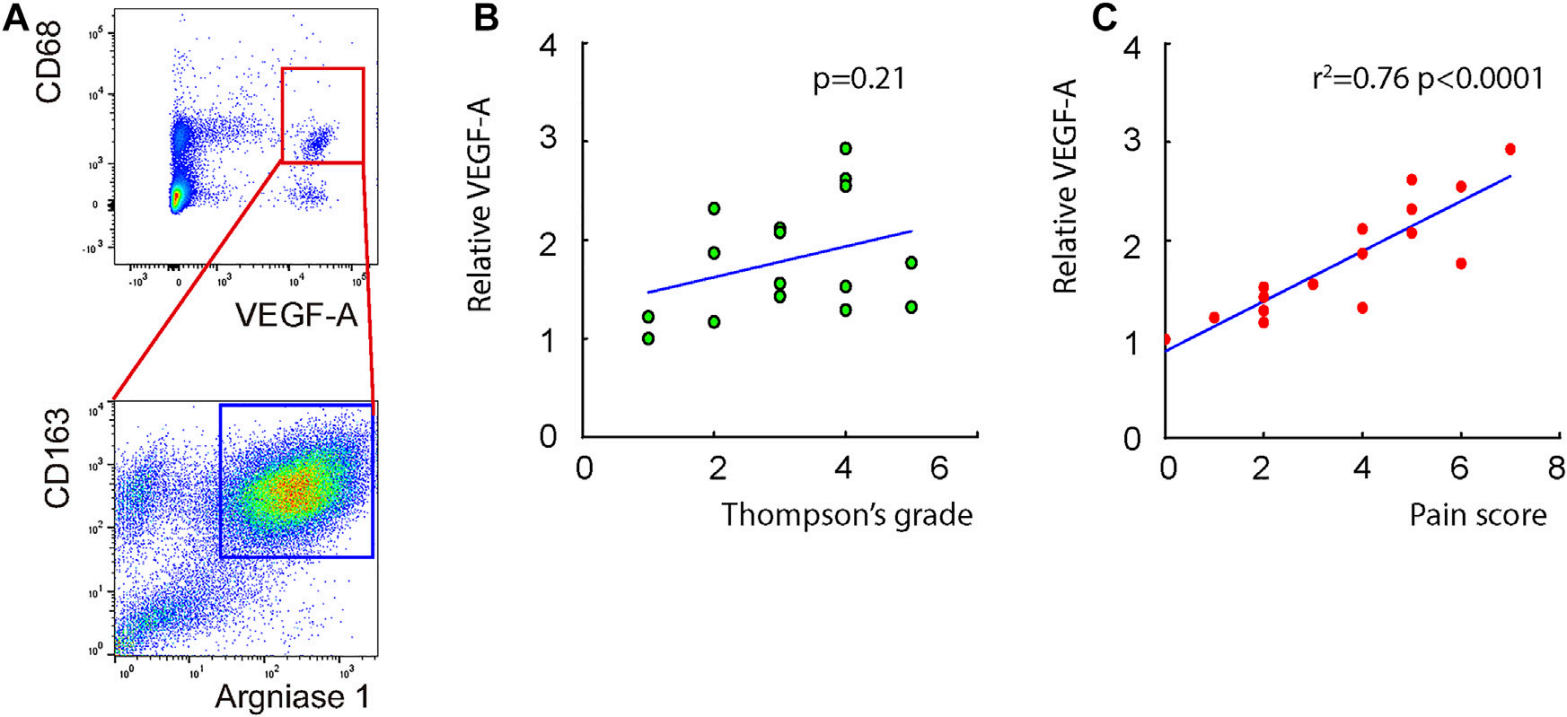
Detection of a VEGF-A-expressing disc macrophage subtype in LDD patients. Disc specimens were analyzed from 16 participants who had different Thompson classification of the degeneration level and pain score. **(A)** The disc specimens were digested into a single cell fraction and subjected to fluorescence activated cell sorting (FACS) for CD68 and VEGF-A. The VEGF+CD68^+^ cells were further analyzed for CD163 and Arginase 1, shown by a representative flow chart. **(B–C)** The VEGF-A transcripts in these VEGF-A+ macrophages were quantified in all 16 specimens. The correlation between VEGF-A transcripts and Thompson classification of the degeneration level [**(B)**, *p* = 0.21] or pain score [**(C)**, *r* ^2^ = 0.75, *p* < 0.0001] was assessed.

Macrophages appeared in the degenerating discs with innervation and angiogenesis, which contribute to the LDD-associated pain. To assess the role of disc macrophages in the LDD-associated pain, we analyzed disc specimens from 16 participants who had different Thompson classification of the degeneration level and pain score (Table 1). The disc specimens were digested into a single cell fraction and subjected to fluorescence activated cell sorting (FACS) for CD68, a pan-macrophage marker, and for VEGF-A, the most potent angiogenetic factor. Interestingly, a fraction of VEGF-A+ cells were detected in CD68^+^ macrophages, and the number of VEGF+CD68^+^ cells was more than the VEGF+CD68^−^cells, suggesting that these VEGF-A+ macrophages were the major source of VEGF-A in the sick discs (Figure 1A). Moreover, further analysis for M2 macrophage markers, CD163 and arginase 1, on these VEGF-A+ macrophages showed that they were M2 macrophages (Figure 1A). Since VEGF-A is expressed in M2d macrophages, these detected VEGF-A+ macrophages should be M2d macrophages. Next, the VEGF-A transcripts in these VEGF-A+ macrophages were quantified in all 16 specimens. The correlation between VEGF-A transcripts and Thompson classification of the degeneration level or pain score was assessed. We did not detect a correlation between VEGF-A transcripts and Thompson classification of the degeneration level (*p* = 0.21; Figure 1B). However, we detected a strong correlation between VEGF-A transcripts and the pain score of the participants (*r* ^2^ = 0.75, *p* < 0.0001; Figure 1C). These data suggest that VEGF-A-expressing disc macrophages may play a role in the LDD-associated pain.

**TABLE 1:** Demographic details of Intervertebral disc donors.

Sample	Sex	Age (years)	Thompson’s grade	Pain score	Relative disc VEGF-A mRNA
1	F	49	I	0	1
2	M	52	I	1	1.22
3	F	55	II	2	1.17
4	M	44	II	4	1.87
5	F	53	II	5	2.32
6	M	52	III	4	2.12
7	M	48	III	2	1.43
8	F	64	III	3	1.56
9	F	42	III	5	2.08
10	M	67	IV	2	1.29
11	F	59	IV	2	1.53
12	F	63	IV	5	2.62
13	M	48	IV	6	2.55
14	F	58	IV	7	2.93
15	F	47	V	4	1.32
16	M	46	V	6	1.77

### Vascular endothelial growth factor A signaling is activated in discs from lumbar disc degeneration

**FIGURE 2:**
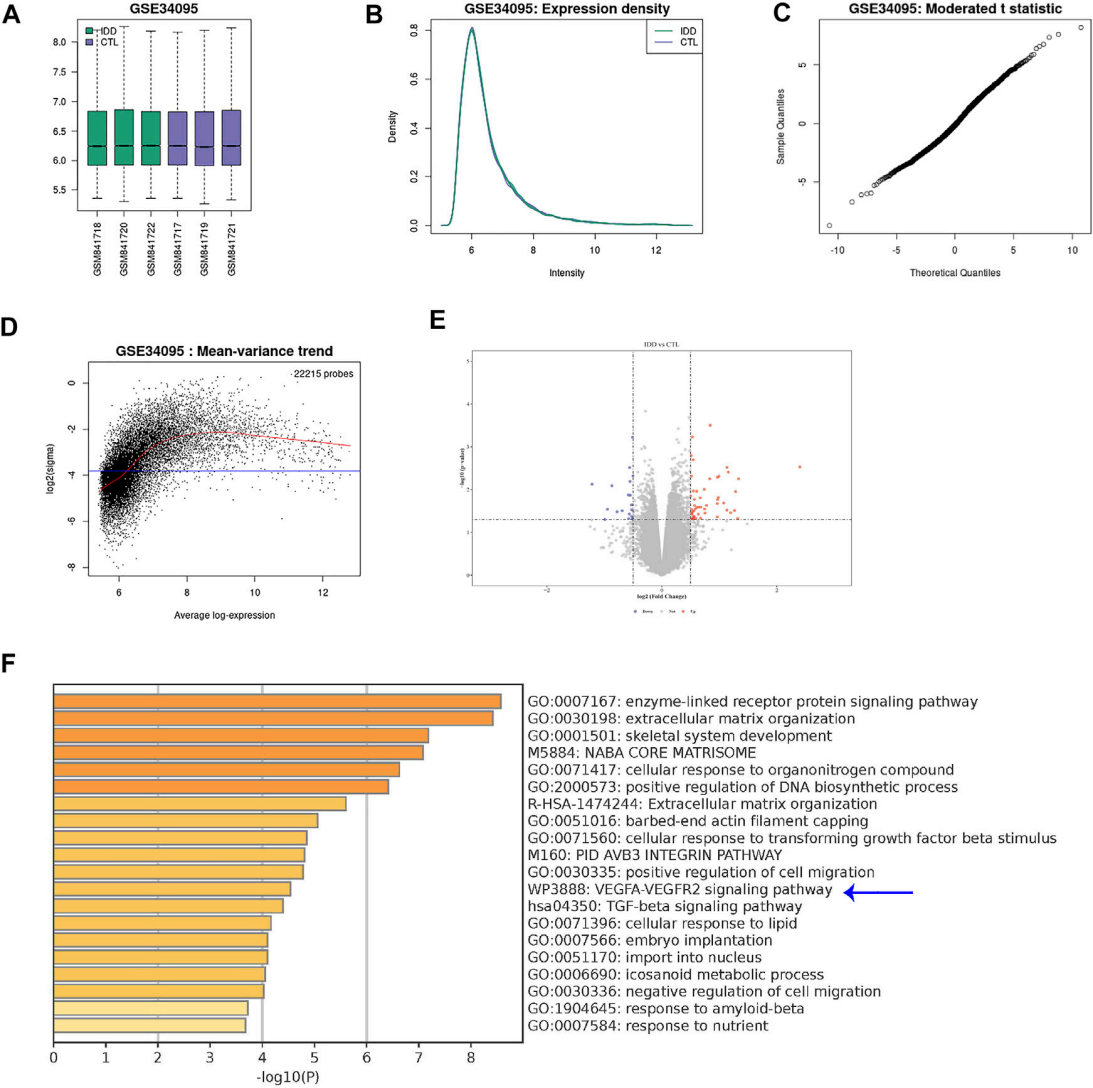
VEGF-A signaling is activated in discs from LDD. A GEO database (GSE34095) including 3 LDD specimens and 3 controls was analyzed. **(A)** Principal component analysis (PCA). **(B)** Expression density. **(C)** Moderated t statistic test. **(D)** Mean-variance trend. **(E)** A volcano map showing differential genes upregulated or downregulated in LDD, compared to controls. **(F)** KEGG pathway analysis showed that VEGF-A signaling pathway (blue arrow) was a major altered one.

Since innervation and angiogenesis are known to contribute to the LDD-associated pain, we analyzed data from public databases to confirm our findings on VEGF-A+ macrophages. A GEO database (GSE34095) including 3 LDD specimens and 3 controls was used. A set of quality controls were performed to validate the data, including principal component analysis (PCA; Figure 2A), expression density (Figure 2B), moderated t statistic test (Figure 2C) and mean-variance trend (Figure 2D). We detected some differential genes upregulated or downregulated in LDD, compared to controls (Figure 2E). Interestingly, KEGG pathway analysis showed that VEGF-A signaling pathway was a major altered one (Figure 2F). Thus, VEGF-A signaling is activated in discs from LDD, consistent with our finding.

### Vascular endothelial growth factor A and angiogenesis -associated genes are the mainly affected ones in discs from lumbar disc degeneration

**FIGURE 3:**
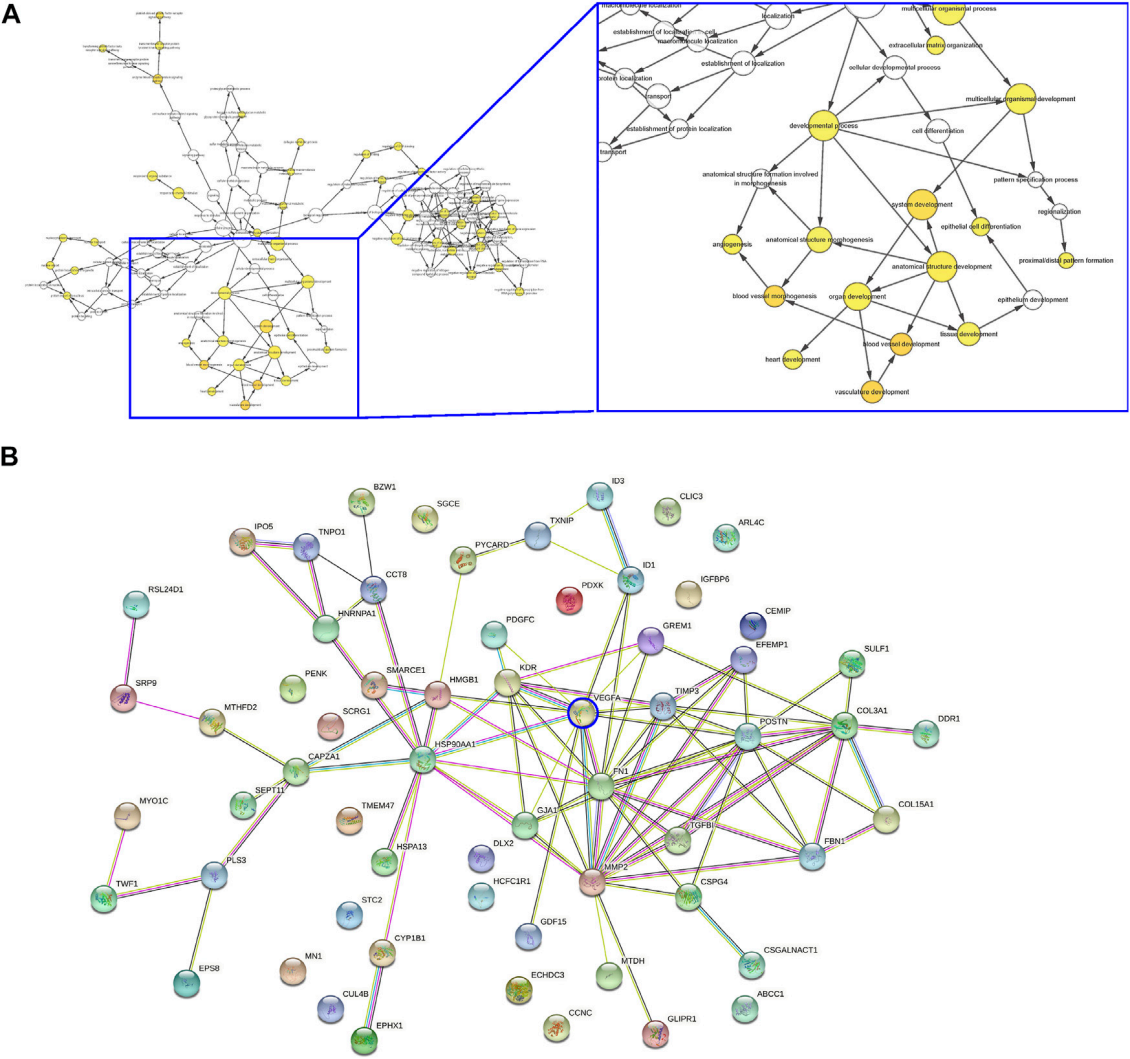
VEGF-A and angiogenesis -associated genes are the mainly affected ones in discs from LDD. **(A–B)** Differential genes in the analysis for GEO34095 were presented for pathway analysis by Cytoscope **(A)** and gene-gene relationship by String **(B)**.

Next, the differential genes were used for pathway analysis by Cytoscope (Figure 3A) and gene-gene relationship by String (Figure 3B) online tools, respectively. Interestingly, the major affected pathways in LDD included many associated with angiogenesis and vascular development (Figure 3A), while the major affected genes in LDD included many associated with VEGF-A signaling and angiogenesis (Figure 3B). Moreover, among these genes, VEGF-A appeared to be a central one in the gene interaction network, while other differential genes with more connectors in the VEGF-A signaling included matrix metalloproteinase 2 (MMP2), Fibronectin 1 (FN1), TIMP Metallopeptidase Inhibitor 3 (TIMP3), Collagen Type III Alpha 1 Chain (COL3A1) and Collagen Type XV Alpha 1 Chain (COL15A1) (Figure 3B). Together, these data suggest that VEGF-A and angiogenesis -associated genes are the mainly affected ones in discs from LDD, again consistent with our finding.

### Generation and validation of AAVs with altered levels of vascular endothelial growth factor A

**FIGURE 4:**
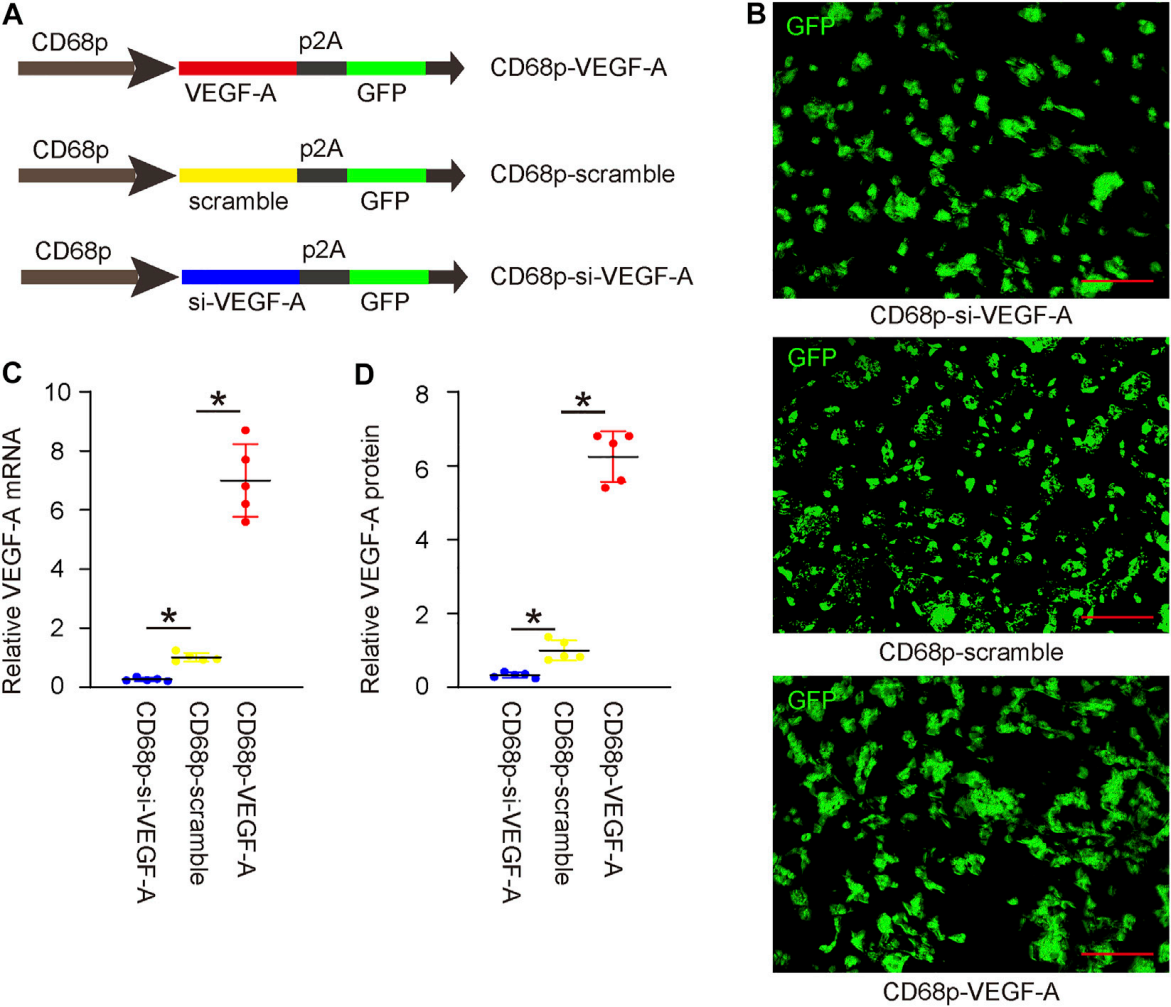
Generation and validation of AAVs with altered levels of VEGF-A. **(A)** AAVs carrying either complete coding sequence for VEGF-A or siRNA for VEGF-A (si-VEGF-A) under a macrophage-specific CD68 promoter were prepared. A scramble sequence was used to replace the transgene to be driven by the CD68 promoter as well. All 3 AAVs contained a GFP reporter to be co-driven under the CD68 promoter using a p2A construct. **(B)** IL-4 primed bone marrow derived macrophages (BMDM) were transduced with 3 AAVs. GFP was shown in cultured cells. **(C)** RT-qPCR on VEGF-A mRNA. **(D)** ELISA on VEGF-A protein. *p < 0.05. N = 5. Scale bars were 100 µl.

In order to alter VEGF-A levels in macrophages and assess its effects on LDD-associated pain, we prepared AAVs that carried either complete coding sequence for VEGF-A (functional for both human and mouse due to similarity of their sequences) or siRNA for VEGF-A (si-VEGF-A) under a macrophage-specific CD68 promoter. A scramble sequence was used to replace the transgene to be driven by the CD68 promoter as well. All 3 AAVs contained a GFP reporter to be co-driven under the CD68 promoter using a p2A construct (Figure 4A). This CD68 promoter has been shown functional for both human and mouse macrophages. IL-4 was used to induce M2 macrophage polarization, after which IL-4 primed bone marrow derived macrophages (BMDM) were used for validation, to mimic the macrophage phenotype detected in LDD. IL-4 was not added to the co-culture, since it may regulate the angiogenesis of endothelial cells by itself (Kotowicz et al., 1996). We found that macrophages were effectively transduced with 3 AAVs with a more than 95% infection efficiency (Figure 4B). Transduction with VEGF-A virus significantly increased VEGF-A levels in macrophages, by RT-qPCR on mRNA (Figure 4C), and by ELISA on protein (Figure 4D). On the other hand, transduction with si-VEGF-A virus significantly decreased VEGF-A levels in macrophages, by RT-qPCR on mRNA (Figure 4C), and by ELISA on protein (Figure 4D).

### Vascular endothelial growth factor A in macrophages promotes angiogenesis and innervation *in vitro*

**FIGURE 5:**
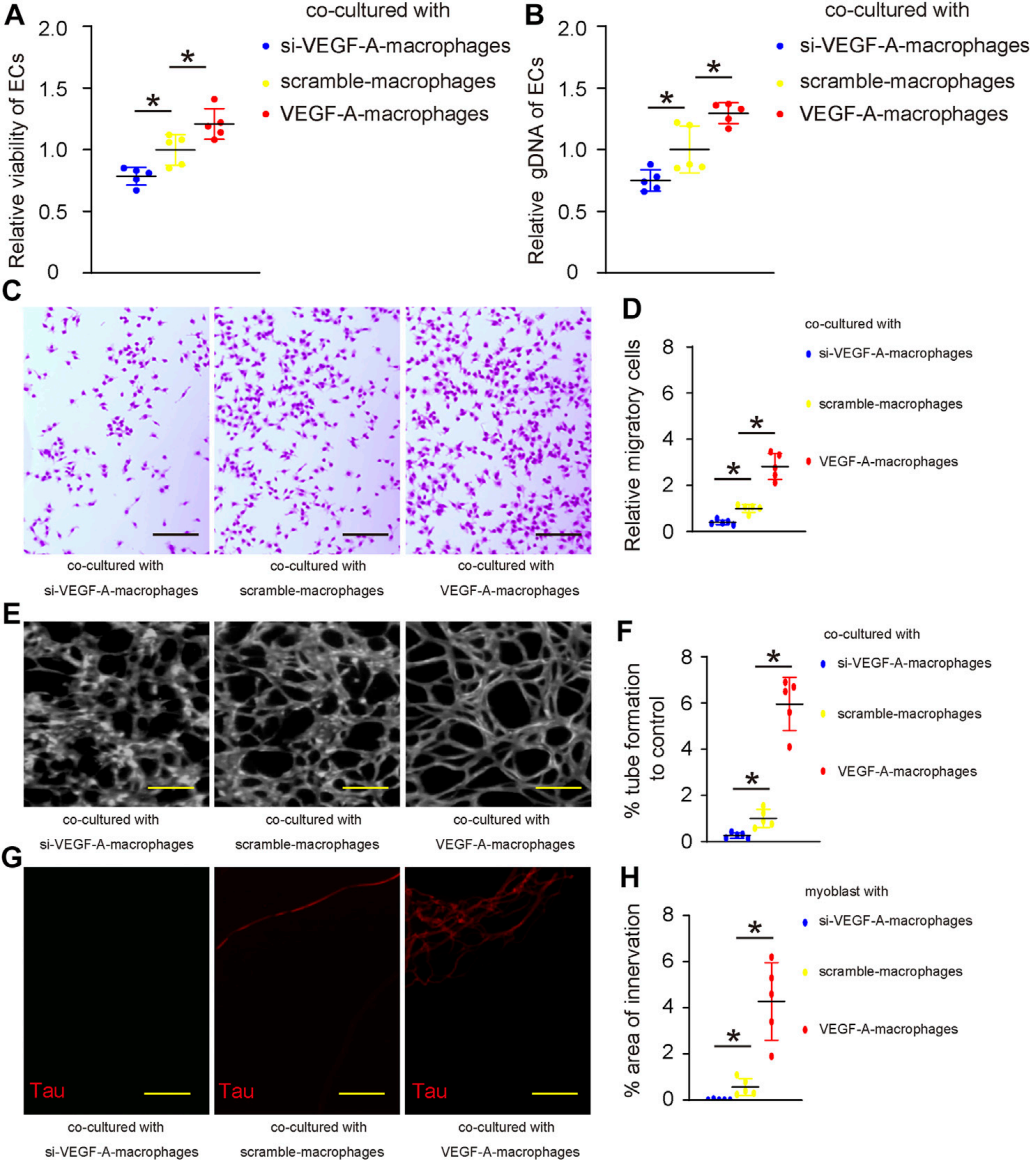
VEGF-A in macrophages promotes angiogenesis and innervation *in vitro*. **(A–F)** VEGF-A, scramble, or si-VEGF-A virus-transduced mouse IL-4-primed BMDM were co-cultured with mouse endothelial cells (ECs). **(A–B)** The growth of ECs was analyzed by a CCK-8 assay **(A)** together with quantification of total genomic DNA content (gDNA) on them **(B)**. **(C–D)** The migratory potential of ECs was assessed, shown by representative images **(C)** and by quantification **(D)**. **(E–F)** The angiogenetic potential of ECs was assessed in a tube formation assay, shown by representative images **(E)** and by quantification **(F)**. **(G,H)** The effects of VEGF-A in macrophages on innervation was assessed in a microfluidic chamber system that allows induction of innervation of embryonic spine cord explant into the co-cultured area for macrophages and skeletal myocytes, shown by representative images for Tau staining in the co-culture area **(G)** and by quantification **(H)**. *p < 0.05. N = 5. Scale bars were 100 µl.

To assess the effects of macrophage VEGF-A on angiogenesis and innervation, we did two *in vitro* experiments. First, VEGF-A, scramble, or si-VEGF-A virus-transduced mouse IL-4-primed BMDM were co-cultured with mouse endothelial cells (ECs). The growth of ECs was analyzed by a CCK-8 assay together with quantification of total genomic DNA content (gDNA) on them. We found that co-culture with VEGF-A-transduced macrophages significantly increased the number of ECs (Figure 5A) and their total gDNA (Figure 5B). On the other hand, co-culture with si-VEGF-A-transduced macrophages significantly decreased the number of ECs (Figure 5A) and their total gDNA (Figure 5B). Thus, VEGF-A in macrophages promotes EC growth in co-culture. Next, the migratory potential of ECs was assessed, showing that co-culture with VEGF-A-transduced macrophages significantly increased the migratory potential of ECs (Figures 5C,D), while co-culture with si-VEGF-A-transduced macrophages significantly decreased the migratory potential of ECs (Figures 5C,D). Thus, VEGF-A in macrophages promotes EC migration in co-culture. Then, the angiogenetic potential of ECs was assessed in a tube formation assay, showing that co-culture with VEGF-A-transduced macrophages significantly increased the tube formation of ECs (Figures 5E,F), while co-culture with si-VEGF-A-transduced macrophages significantly decreased the tube formation of ECs (Figures 5E,F). Thus, VEGF-A in macrophages promotes angiogenetic potential of ECs in co-culture. Finally, the effects of VEGF-A in macrophages on innervation was assessed in a Microfluidic chamber system that allows induction of innervation of embryonic spine cord explant into the co-cultured area for macrophages and skeletal myocytes. Our data showed that significantly more innervation occurred in the co-cultured area for myocytes and VEGF-A-transduced macrophages, while significantly less innervation occurred in the co-cultured area for myocytes and si-VEGF-A-transduced macrophages (Figures 5G,H). Together, VEGF-A in macrophages promotes both angiogenesis and innervation *in vitro*.

### Vascular endothelial growth factor A suppression in macrophages reduces lumbar disc degeneration-associated pain *in vivo*

**FIGURE 6:**
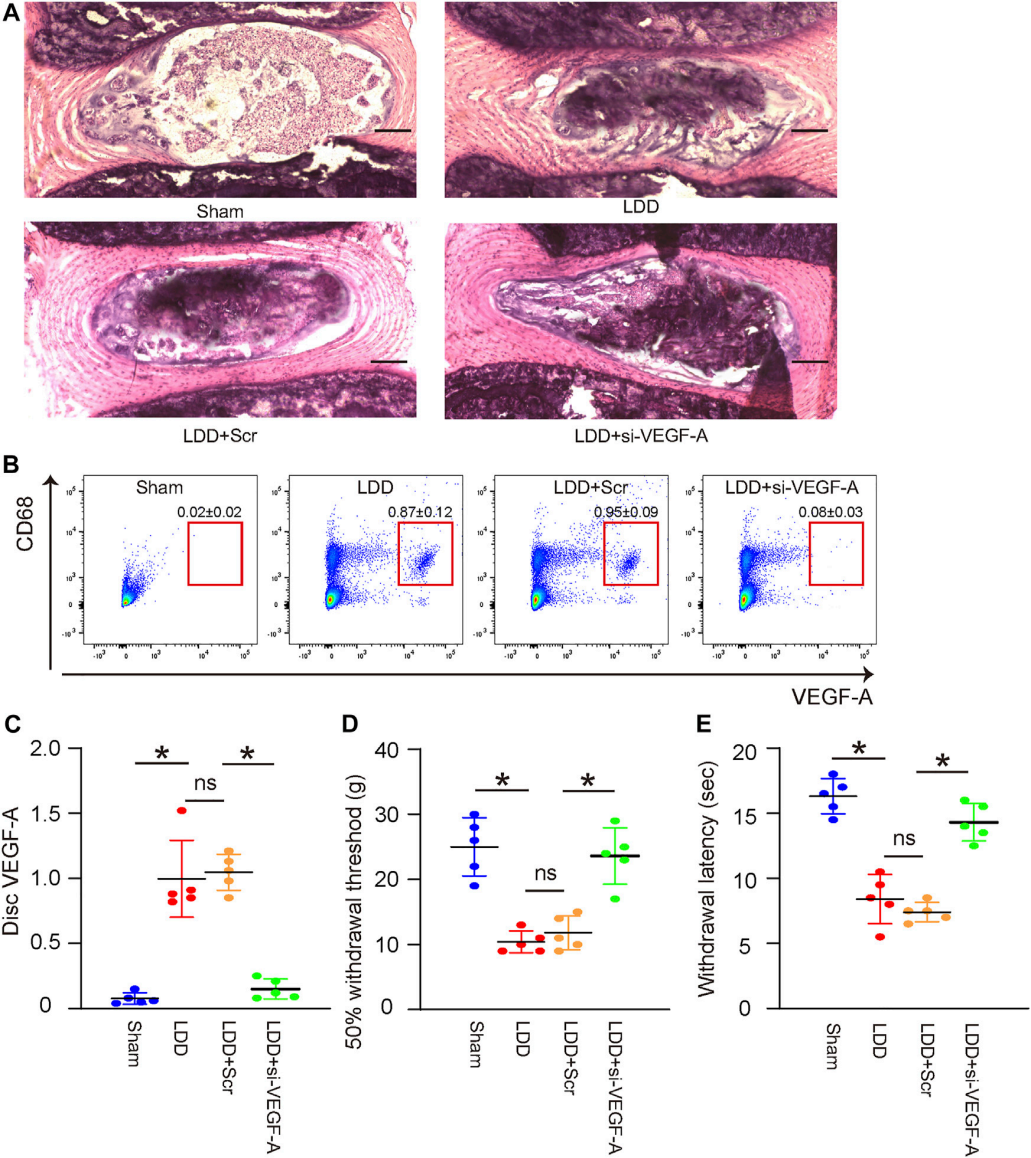
VEGF-A suppression in macrophages reduces LDD-associated pain *in vivo*. The effects of VEGF-A suppression in macrophages on LDD-associated pain were examined in a mouse model for LDD *in vivo*. Four groups of mice were included in this experiment. Group 1, mice received sham operation (Sham); Group 2, mice received LDD induction; Group 3, mice received LDD induction and simultaneous orthotopic injection of scramble-AAVs; Group 4, mice received LDD induction and simultaneous orthotopic injection of si-VEGF-A-AAVs. Mice were analyzed 4 weeks after treatments. **(A)** Representative H&E staining images. **(B)** CD68 and VEGF-A expression of disc macrophages were analyzed by FACS. **(C)** RT-qPCR for disc VEGF-A. **(D–E)** A Von Frey filament test for pain evaluation, shown by mechanically induced withdrawal threshold **(D)** and by thermally induced withdrawal latency of the paw **(E)**. *p < 0.05. ns, no significance. N = 5.

The effects of VEGF-A suppression in macrophages on LDD-associated pain were examined in a mouse model for LDD *in vivo*. Four groups of mice were included in this experiment. Group 1, mice received sham operation (Sham); Group 2, mice received LDD induction (LDD); Group 3, mice received LDD induction and simultaneous orthotopic injection of scramble-AAVs (LDD+Scr); Group 4, mice received LDD induction and simultaneous orthotopic injection of si-VEGF-A-AAVs (LDD+si-VEGF-A). Mice were analyzed 4 weeks after treatments. The generation of surgical development of LDD was confirmed by histology (Figure 6A; Table 2). The effects of these treatments on disc macrophages were analyzed by FACS (Figure 6B). Hardly any macrophages (including VEGF-A+ macrophages) were detected in discs from sham mice, while macrophages (including VEGF-A+ macrophages) were detected in discs from LDD mice (Figure 6B). The number of VEGF-A+ macrophages, but not total number of macrophages, significantly decreased in discs from LDD mice that had received si-VEGF-A-AAVs (Figure 6B). Moreover, the total disc VEGF-A transcripts significantly decreased in LDD mice that had received si-VEGF-A-AAVs (Figure 6C). These data validated the effects of AAVs on disc macrophages. The mechanical and thermal pain was evaluated by a Von Frey filament test, showing significant improvement in mechanically induced withdrawal threshold (Figure 6D) and in thermally induced withdrawal latency of the paw (Figure 6E) in LDD mice that had received si-VEGF-A-AAVs. These data suggest that VEGF-A suppression in macrophages reduces LDD-associated pain *in vivo*.

**TABLE:** 2Histological LDD degenerative score.

Groups	Degenerative score (mean ± SD)	*p* Value
Sham	6.4 ± 0.5	
LDD	16.3 ± 1.9	<0.05 vs. sham
LDD+Scr	14.9 ± 1.3	<0.05 vs. sham
LDD+si-VEGF-A	14.2 ± 1.6	<0.05 vs. sham

Groups: Group 1, mice received sham operation and injection of empty solvent of equal volume (Sham); Group 2, mice received LDD induction by surgical removal of the spinal muscles, ligaments from supraspine and intraspine and posterolateral halves of the bilateral zygapophysial joints and injection of empty solvent of equal volume (LDD); Group 3, mice received LDD induction and simultaneous orthotopic injection of 100 µl 10^11^ scramble-AAVs (LDD+Scr); Group 4, mice received LDD induction and simultaneous orthotopic injection of 100 µl 10^11^ si-VEGF-A-AAVs (LDD+si-VEGF-A). Mice were analyzed 4 weeks after treatments to determine the pathological changes.

## Discussion

Chronic inflammation is a key and persistent factor coordinating the progressive development of disc degeneration, while macrophages are known to play a pivotal role in the process (Kawakubo et al., 2020). The most important symptom for LDD is the back pain, which results from angiogenesis and innervation in the avascular discs (Yang et al., 2019). Indeed, a more extensive disc innervation has been detected in the severely degenerated human lumbar discs compared with normal controls, as the source of the discogenic pain (Coppes et al., 1997). Disc angiogenesis and innervation often occur simultaneously (Binch et al., 2015) and have been found induced by inflammatory cytokines, e.g., IL-1β (Lee et al., 2011). However, it is not known whether disc macrophages play a role in the angiogenesis and innervation during LDD.

Here, we showed that a macrophage subtype that expresses VEGF-A, M2d, was present in the discs from patients. VEGF-A is expressed in all macrophages, including M1 and M2a-d, but at different levels. The levels of VEGF-A in M2d are the highest among all macrophage subtypes, which is the reason why M2d macrophages are also called angiogenic macrophages (Wang et al., 2010; Nikovics et al., 2020). All the macrophage classification is not actually very rigid. It is now known that polarization of macrophages into the precise definition of “M1” or “M2” macrophages rarely occurs. Instead, macrophage typically polarize into a wide spectrum of phenotypes that exhibit distinct gene and protein expression patterns (Nahrendorf and Swirski, 2016). This broad range of differentiation pattern allows macrophages to perform diverse tasks throughout the body (Nahrendorf and Swirski, 2016). The further sub-classification of M2 into M2a, M2b, M2c, and M2d is also not rigid. There are hardly any macrophages that could be strictly named as perfect M2a or M2b or M2c or M2d macrophages. It depends on how close of the epigenetic profile of one macrophage to any of these macrophage subpopulations. M2d macrophages could be also said as angiogenetic M2 macrophages with high expression of VEGF-A.

Here, the macrophage-derived VEGF-A appeared to be the major source of total VEGF-A in the degenerating discs. Therefore, a strong correlation of macrophage-derived VEGF-A with pain but not with degeneration level could result from the effects on angiogenesis and innervation, rather than on the fibrosis of the discs or the apoptosis of disc cells. Indeed, this hypothesis was confirmed by *in vitro* co-culture experiments examining the effects of macrophage-derived VEGF-A on EC and nerve growth. In those experiments, we modified VEGF-A levels on IL-4 primed M2 macrophages, rather than naïve macrophages or M1 macrophages, since what we detected in the human degenerating discs were M2 macrophages expressing VEGF-A. This aim for this experimental design was to mimic the condition in the human disease to the maximum.

We detected a network including many differential genes upstream or downstream of VEGF-A in the LDD discs compared to normal controls. Among those genes, we specifically found that MMP2, FN1, TIMP3 COL3A1, and COL15A1. Interestingly, MMP2 and TIMP3 are associated with extracellular matrix degradation, while FN1, COL3A1, and COL15A1 are associated with fibronectin and collagen formation (Wang et al., 2018). Hence, the activation of VEGF-A signaling in discs may not only trigger angiogenesis, but also induce fibrosis that contribute to LDD. Moreover, VEGF-A in macrophages may later induce a M2c fibrotic phenotype to promote the degenerating process of the discs. Therefore, the suppression of the angiogenetic potential of disc macrophages could also reduce polarization of M2c macrophages in addition to its suppression on M2d polarization, both improving the outcome of the therapy (Hou et al., 2020). This possibility was not further investigated here. Moreover, only one end one was analyzed in the current study, which is a limitation. MRI monitoring at different time points after treatment could also provide useful information (Leckie et al., 2012). These points could be further addressed in the future studies.

To summarize, here we present strong data to demonstrate a critical role of VEGF-A-expressing M2d macrophages in the development of LDD-associated pain. We also provided a therapeutic strategy to release LDD-associated pain through suppressing VEGF-A in disc macrophages.

## Data Availability

The original contributions presented in the study are included in the article/supplementary material, further inquiries can be directed to the corresponding author.
